# A Systematic Approach towards Optimizing a Cohabitation Challenge Model for Infectious Pancreatic Necrosis Virus in Atlantic Salmon (*Salmo salar* L.)

**DOI:** 10.1371/journal.pone.0148467

**Published:** 2016-02-19

**Authors:** Hetron Mweemba Munang’andu, Nina Santi, Børge Nilsen Fredriksen, Knut-Egil Løkling, Øystein Evensen

**Affiliations:** 1 Norwegian University of Life Sciences, Faculty of Veterinary Medicine and Biosciences, Department of Basic Sciences and Aquatic Medicine, Section of Aquatic Medicine and Nutrition, Ullevålsveien 72, Oslo, Norway; 2 AquaGen, Trondheim, Norway; 3 PHARMAQ AS, Harbitzalléen 2A, 0275, Oslo, Norway; INRA, FRANCE

## Abstract

A cohabitation challenge model was developed for use in evaluating the efficacy of vaccines developed against infectious pancreatic necrosis virus (IPNV) in Atlantic salmon (*Salmo salar* L) using a stepwise approach. The study involved identifying a set of input variables that were optimized before inclusion in the model. Input variables identified included the highly virulent Norwegian Sp strain NVI015-TA encoding the T_217_A_221_ motif having the ability to cause >90% mortality and a hazard risk ratio of 490.18 (p<0.000) for use as challenge virus. The challenge dose was estimated at 1x10^7^ TCID_50_/mL per fish while the proportion of virus shedders was estimated at 12.5% of the total number of fish per tank. The model was designed based on a three parallel tank system in which the Cox hazard proportional regression model was used to estimate the minimum number of fish required to show significant differences between the vaccinated and control fish in each tank. All input variables were optimized to generate mortality >75% in the unvaccinated fish in order to attain a high discriminatory capacity (DC) between the vaccinated and control fish as a measure of vaccine efficacy. The model shows the importance of using highly susceptible fish to IPNV in the optimization of challenge models by showing that highly susceptible fish had a better DC of differentiating vaccine protected fish from the unvaccinated control fish than the less susceptible fish. Once all input variables were optimized, the model was tested for its reproducibility by generating similar results from three independent cohabitation challenge trials using the same input variables. Overall, data presented here show that the cohabitation challenge model developed in this study is reproducible and that it can reliably be used to evaluate the efficacy of vaccines developed against IPNV in Atlantic salmon. We envision that the approach used here will open new avenues for developing optimal challenge models for use in evaluating the efficacy of different vaccines used in aquaculture.

## Introduction

Infectious pancreatic necrosis (IPN) is a highly contagious infectious viral disease of juvenile salmonids known to cause high economic losses in aquaculture. It is caused by infectious pancreatic necrosis virus (IPNV), a member of the genus *aquabirnavirus*, of the *Birnaviridae* family [1]. Although efficacious vaccines have been developed, IPN has continued to ravage the salmon industry [2]. One of the major constraints to developing highly efficacious vaccines against IPN in salmonids has been the general lack of a reliable challenge model required for evaluating vaccine efficacy [3,4]. As pointed out by different scientists [3–5], a reliable challenge model is a prerequisite to designing successful vaccine efficacy trials and as such optimization of challenge models is an integral part of vaccine development in aquaculture. However, to develop a reliable challenge model several input variables have to be generated and each variable has to be optimized in order to ensure that it contributes positively to the overall performance of the model. Although we have recently documented the use of a cohabitation challenge model in evaluating the efficacy of IPN vaccines in Atlantic salmon (*Salmo salar* L) [6–8], steps leading to its optimization have not been documented.

Hence, in the present study, we put together several studies that show a stepwise approach into developing a reproducible cohabitation challenge model for IPN in Atlantic salmon. To attain this, we set a cutoff limit of ≥75% post challenge cumulative mortality in the unvaccinated control fish for the optimization of all input variables to enable us attain a wide discriminatory capacity between the vaccinated and control fish as a measure of vaccine efficacy. Using a systematic approach, we demonstrate the importance of identifying different input variables required to develop a reliable and reproducible cohabitation challenge model as well as the importance of optimizing each input variable to ensure that it attains the established ≥75% post challenge mortality benchmark in the control fish. We anticipate that the approach used in this study shall open new avenues for optimizing challenge models used for evaluating the efficacy of different fish vaccines used in aquaculture.

## Materials and Methods

### Cells, viruses and vaccine

All viruses were propagated on rainbow trout gonad (RTG-2, ATCC CC-55) cells, which is a cell-line known not to cause mutations on the deduced virulence motif [9,10]. This cell line was cultured at 15°C in Leibowitz L-15 media supplemented with 10% fetal serum (FBS) (Sigma), 2% L-glutamine (Sigma) and gentamicin (sigma) 25μg/mL. Viruses used to identify the challenge strain were previously isolated from field outbreaks in Norway as reported by Santi et al [10]. Construction of recombinant viruses rNVI015-TA, rNVI015PA and rNVI015PT to verify the virulence motifs by reverse genetics followed by recovery of the infectious clone by plaque purification assays has been previously described by Santi et al [9,10]. Genebank accession numbers for the viruses used in the study are shown in Table 1. The vaccines used were produced by PHARMAQ AS for commercial use by fish farmers. The vaccines were bought from pharmaceutical retailers and used according to manufacturer’s recommendations.

**Table 1:** Post challenge survival proportions and hazard ratios of different IPNV isolates used for selecting the challenge virus strain.

Classification	virus strain	Motif	*N*	PCSP (%)	Hazard ratio	Z	P>Z	95%CI	
Virulent strains	NV1011	T_217_A_221_	187	10.16%	483.00	6.16	0.000	67.60	3450.95
	NVI013	T_217_A_221_	188	10.11%	438.87	6.06	0.000	61.42	3135.61
	NVI015	T_217_A_221_	182	8.24%	490.18	6.17	0.000	68.60	3502.64
	NVI020	T_217_A_221_	189	12.17%	401.37	5.98	0.000	56.18	2867.37
	NVI023	T_217_A_221_	191	15.71%	384.28	5.93	0.000	23.78	2745.76
Moderate strain	NVI010	P_217_A_221_	185	52.43%	101.71	4.6	0.000	14.17	730.17
Avirulent strains	NVI001	P_217_T_221_	187	98.40%	3.07	0.97	0.331	0.32	29.55
	NVI016	P_217_T_221_	186	96.78%	6.11	1.68	0.094	0.74	50.77
	NVI024	P_217_T_221_	194	96.88%	7.95	1.95	0.052	0.99	63.53

PCSP = Post challenge survival proportions; Virus strain = NVI designate Norwegian Veterinary Institute while the digits represent the isolate reference number; Motif = deduced amino acid positions linked to virulence on amino acid position 217 and 221 the VP2 capsid of IPNV; *N* = number of fish challenged for each virus strain; PCSP = post challenge survival proportion (percent).

### Study fish

Atlantic salmon, AquaGen AS breed, whose susceptibility to IPNV was known by genetic selection were used in the study. Fish experiments were carried out at VESO Vikan in Namsos and Havbruksstasjonen i Tromsø (HiT) (Tromsø Aquaculture Research station), Norway. Fish were fed commercial feed (Skretting, Norway) *ad libitum* in all studies.

### Experimental designs

Six different studies were carried out in a stepwise approach to identify the different input variables required to develop a cohabitation challenge model for IPNV in Atlantic salmon. Each variable was optimized to attain a benchmark of ≥75% mortality in the unvaccinated control fish as a cutoff limit. The experiments/procedures reported herein have been conducted in accordance with the laws and regulations controlling experiments/procedures in live animals in Norway, i.e. the Animal Welfare Act of December 20th 1974, No 73, chapter VI sections 20–22 and the Regulation on Animal Experimentation of January 15th 1996. In addition, Norway has signed and ratified The European Convention for the protection of Vertebrate Animals used for Experimental and other Scientific Purposes of March 18th 1986. The Norwegian legislation conforms in all respects with the basic requirements of this Convention and guidelines prepared in pursuance of it. The National Animal Research Authority (NARA) and the IACUC committee of the University of Tromsø Research Station have approved the entire study. The animals were monitored a minimum of three times daily for signs of disease (particularly post challenge) and fish with clinical signs like slow swimming, distorted movements or color changes were collected and euthanized with a shart blow to the head (standard method). Unexpected deaths did not occur and vaccination was carried out under anesthesia (Finquel, 300mg/L of water) for all vaccination groups.

#### Study I—Choice of challenge virus strain

Nine isolates of IPNV previously obtained from field outbreaks in Norway were tested for their virulence in a highly susceptible strain of Atlantic salmon fry to IPNV infection in order to identify a strain able to cause ≥75% mortality in the challenged fish. A total of 1878 fry at an average weight of 0.16 grams were used and were allocated into 10 different groups based on the nine isolates obtained from the field plus one control group. The numbers of fish allocated for each isolate is shown in Table 1 and each group was further subdivided into four tanks with each tank having approximately 45–47 fish. Once allocated to their respective tanks, fish were left to acclimatize for 1 week. Thereafter, they were challenged by immersion at 10^4^ TCID50/mL^-1^ using the nine isolates obtained from the field while the control group was exposed to phosphate buffered saline (PBS) by immersion. After challenge, mortality was recorded daily until cessation. Samples collected to verify the presence of IPNV comprised of the pancreas, liver and headkidney organs from 12 fish from each group. Headkidney samples were used for virus characterization while the liver and pancreas samples were preserved in 10% formalin for histopathology.

#### Study II—Estimation of challenge dose

The second study focused on optimization of the challenge dose using the challenge virus strain identified in study I above. Two groups of 90 fish each obtained from an Atlantic salmon strain highly susceptible to IPNV infection were used in this study. In each group, 45 fish were vaccinated using a commercial vaccine (PHARMAQ AS) while another 45 were injected with PBS to serve as unvaccinated control fish. The vaccinated and control fish were put in one tank and left for smoltification during which time vaccinated fish developed immune responses to vaccination. At 840 degree days (dd), fish were challenged using a high challenge dose (HC_dose_) of 1 x10^7^ TCID_50_/mL or a low challenge dose (LC_dose_) 1 x10^5^ TCID_50_/mL. The choice of challenge doses was based on challenge doses previously used by other scientists [11]. After challenge, mortality was recorded daily until cessation. Blood samples were collected at 330 dd and at 840 dd after vaccination to evaluate the antibody responses during the immune induction period. Head kidney samples were collected from 12 fish from each group to compare the virus carrier state in fish exposed to the two challenge doses at 10 weeks post challenge.

#### Study III- Estimation of the proportion of virus shedders

To optimize the proportion of virus shedders needed to induce ≥ 75% mortality in the un-vaccinated fish and to determine whether increasing the proportion of virus shedders would increase or accelerate the onset of mortality in the cohabitees, three groups each comprising of 90 Atlantic salmon smolts obtained from a strain highly susceptible to IPNV infection were challenged by cohabitation using different shedder ratios. Group-I was allocated 10% (9/90 fish), group-II 20% (18/90 fish) and group-III 30% (27/90 fish) IPNV positive shedders injected with the challenge virus strain identified in study I using a challenge dose determined in study II. Mortality in the IPNV injected shedders and cohabitees was recorded daily post challenge until cessation.

#### Study IV—Susceptibility of the study fish to IPNV infection

A total of 180 Atlantic salmon were used to compare the susceptibility of different strains of Atlantic salmon to IPNV infection and identify a susceptible strain that would yield ≥75% mortality in the unvaccinated cohabitees. One group (N = 90) obtained from a parent stock genetically bred to be highly susceptible to IPNV infection was designated as HS_strain_ while another group (N = 90) generated from a parent stock that was less susceptible to IPN infection was designated as LC_strain_. In each group, 45 fish were vaccinated using a commercial vaccine (PHARMAQ AS) while the control group was injected with PBS. After vaccination, fish were subjected to smoltification during immune induction. At 820 dd, fish were challenged using the highly virulent challenge strain identified in study-I at the concentration established in study-II and a proportion of virus shedders established in study-III. Thereafter, mortality was recorded daily during the challenge period. Blood samples were collected at 350 and 820 dd post vaccination.

#### Study V–Design of challenge model design based on statistical analyses

In conformity with the European Medicine Agency (EMA) requirements [12], which states that at least two parallel tanks should be used for challenge trials to allow for statistical evaluation of inter-tank variations and that the sample size should allow the results to be statistically significant and clinically reliable, the cohabitation challenge model used in this study was designed to use a three parallel tank system. The Cox hazard proportional (PH) regression model was used to estimate the minimum number of fish required to show statistical significance and clinical reliability of the challenge model. The statistical power function used for sample size estimation was set at 80%, 95% confidence limits (CI) and hazard ratio (HR) at 0.5 based on input variables commonly used in clinical trials in higher vertebrates [13–15]. All computations were carried in STATA version 10 (www.stata.com) and once the sample size estimates were determined the number of fish required in a three parallel tank systems were vaccinated and put together with the unvaccinated control fish were in each tank. Thereafter, fish were left for smoltification during immune induction and were later challenged using the challenge virus strain identified in study-I at the challenge dose established in study-II and proportion of virus shedders established in study-III using a susceptible strain of fish identified in study IV. Data obtained from this study was used to test the consistency of the three parallel tank system by showing that fish kept separately in different independent tanks produced similar results by yielding ≥75% mortality in control fish in all three tanks.

#### Study VI—Testing the reproducibility of the model

Four independent cohabitation challenge trials were carried out using the input variables optimized in steps I-V outlined above. Data from this study was used to test the reproducibility of the model by showing that control fish in all the four trials had ≥75% mortality.

### Virus isolation and sequencing

Headkidney samples collected from infected fish were homogenized in transport media using a stomacher (Seward LtD, USA) followed by centrifugation at 2500 rpm for 10 min. Thereafter, 0.1mL was inoculated on RTG-2 cells on 24 wells plates to final dilutions of 1% and 0.1%. The plates were incubated at 15°C for 7 days after which 0.1 mL supernatant was inoculated on a second monolayer of confluent cells for a second passage. The final reading was carried out after the second passage and results were scored based on presence (+) or absence (-) of cytopathogenic effect (CPE). Supernatants from the second passage were used for RNA extraction using the RNAeasy mini kit based on manufacturer’s recommendations (Qiagen, Hilden, Germany). Thereafter RT-PCR was carried out using the A-sp500F and sp1689R primers [10]. PCR products were sequenced to characterize the virus genome and to identify the virulence motif involved.

### Enzyme linked immunosorbent assay (ELISA)

Determination of antibody levels was carried by ELISA as described previously [6]. All viral antigens were used at 1 x 10^5^ TCID_50_/mL, while plasma samples were diluted at 1:50 in PBS.

### Histopathology and immunohistochemistry

All formalin-fixed samples were processed and embedded in paraffin wax and thereafter, histopathology and immunohistochemistry were carried out as previously described [16,17].

### Statistical analysis

The Kaplan Meyer’s (KM) survival analysis was used to generate post challenge survival proportions (PCSPs) [18] while the Cox PH regression model [19] was used to estimate the relative risk of fish dying due to post challenge IPNV infection. All tests were considered significant at p<0.05 and 95%CI.

## Results

Development and optimization of the cohabitation challenge model used in this study was carried out in a stepwise approach outlined below.

### Selection of the challenge virus strain

Table 1 classifies the viral isolates used for the selection of the challenge strain into three categories. Fish exposed to isolates encoding the P_217_T_221_ motif had high PCSP in the range of 96.78%–98.40%. The risk of fish exposed to the P_217_T_221_ motif dying was 12 times higher relative to the unchallenged control fish and their chances of survival was >94% indicating that these isolates did not pose a significant risk of causing high mortality in infected fish. The second category comprised of strain NVI010, which encoded the P_217_A_221_ motif that had a moderately low PCSP (52.43%, p<0.000) with a moderately high relative risk (HR = 101.71, 95% CI 14.17–730.17) of causing mortality in the unvaccinated fish. The final category comprised of isolates encoding the T_217_A_221_ motif that caused high mortality leading to low PCSPs (8.24%–15.71%). Fish challenged using these isolates were >380 times (Table 1) at risk of dying relative to the unchallenged fish showing that isolates encoding the T_217_A_221_ motif were highly lethal and were by far better candidates for use as challenge virus than isolates encoding the P_217_T_217_ and P_217_A_221_ motifs. As a result, strain NVI015, which had the lowest PCSP (8.17%, *n* = 182), and being the most potent isolate having the highest relative risk (HR = 490.18, 95%CI 68.60–3502.64) of causing mortality in infected fish, was chosen for use as challenge virus in the next optimization steps. The chosen strain was designated as NVI015-TA (NVI = Norwegian Veterinary Institute, 015 = isolate number, and TA = T_217_A_221_ motif) based on the nomenclature outlined in Table 1.

### Optimization of the challenge dose

**Fig 1:**
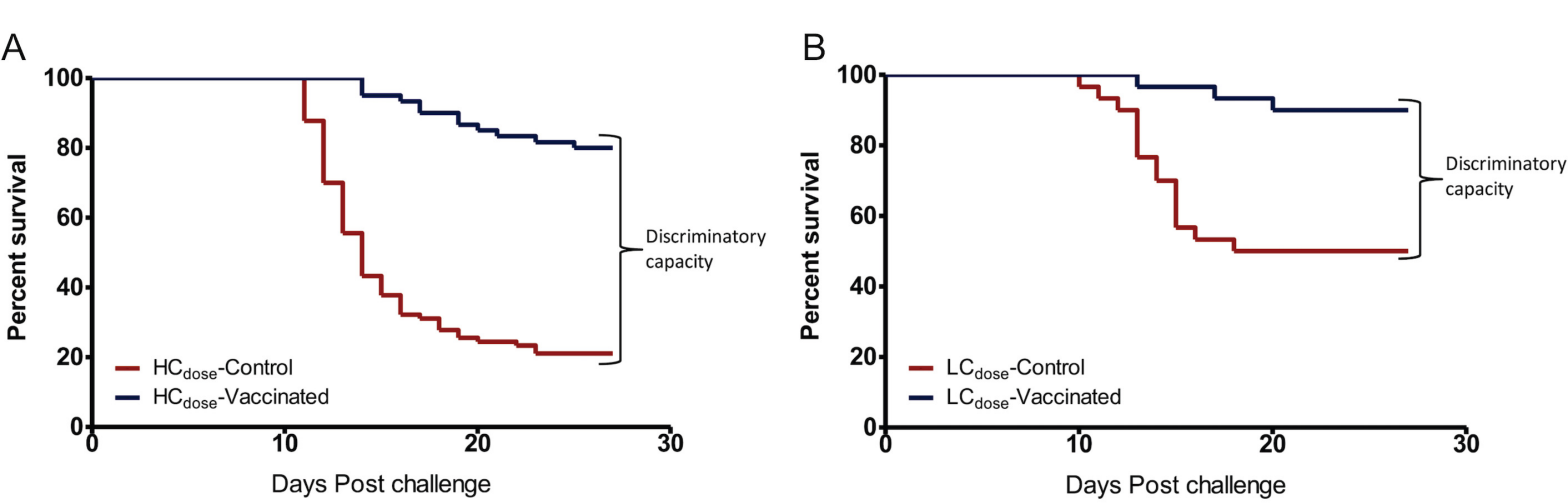
High and low virus challenge dose. Kaplan Meyer’s (KM) survival analysis of Atlantic salmon challenged using a high and low challenge dose carried out using strain NVI015-TA (Study II). (A) KM survival analysis for the high challenge dose (HC_dose_), 1x10^7^ TCID_50_/fish. (B) KM survival analysis for the low challenge dose (LC_dose_), 1x10^5^ TCID_50_/fish. Fig 1A shows a wider discriminatory capacity (DC>58%) compared to Fig 1B (DC = 40%) between the vaccinated and unvaccinated control fish.

Once the challenge virus strain was identified, the next step was to establish the challenge dose for use in the cohabitation model. Fish exposed to the LC_dose_ (1 x 10^5^TCID_50_/fish) had higher PCSPs both for the vaccinated and control fish than fish exposed to the HC_dose_ (1 x 10^7^TCID_50_/fish) (Table 2 and Fig 1). The difference was higher in the control fish in which 50% of fish exposed to the LC_dose_ survived lethal challenge using strain NVI015-TA while only 22.11% survived the challenge using the HC_dose_. These data show that only the HC_dose_ had >75% mortality in control fish that conforms with the cutoff limit set for optimizing the challenge model unlike the LC_dose_ that had 50% mortality that was lower than the cutoff limit of ≥75% mortality. As a result, the ability of the HC_dose_ to differentiate the level of vaccine protection between the vaccinated and control fish was 18.89% higher than the LC_dose_.

**Table 2:** Comparison of post challenge survival proportions and infections of the high and low challenge dose of strain NVI015-TA.

Parameters	Category	Low challenge dose (LC_dose_)	High challenge dose (HC_dose_)	Comparative differences between HC_dose_ and LC_dose_
Post challenge survival proportions (PCSP)	Vaccinated PCSP	90.00%(*N* = 45)	80.00%(*N* = 45)	10.00%
	Unvaccinated PCSP	50.00%(*N* = 45)	21.11%(*N* = 45)	29.89%
	DC	40.00%	58.89%	18.89%
	HR	5.44%	5.89%	0.45%
	95%CI	2.095%–14.10%	3.72%–9.35%	
	P-value	P<0.000	P<0.000	
Post challenge infection (PCI) (10 wpc)	Unvaccinated	100.0% (*N* = 12)	100.0% (*N* = 12)	00.0%
	vaccinated	0.00% (*N* = 12)	44.4% (*N* = 12)	44.4%
	DC	100.0%	55.6%	44.4%
	Relative risk			

PCSP = Post challenge survival proportions; DC = discriminatory capacity calculated as DC = PCSP(vaccinated fish–unvaccinated fish) or PCI(unvaccinated fish–vaccinated fish); HR = Hazard ratio (risk ratio); wpc = weeks post challenge; *N* = total number of fish.

In addition to PCSPs, we analyzed the levels of post challenge virus infection caused by the two challenge doses in vaccinated fish. Table 2 show that vaccinated fish challenged using the LC_dose_ had no virus detected in headkidney samples at 10 weeks post challenge showing that these fish were 100% (*n* = 12) protected against post challenge infection. On the contrary, 44.4% of the fish challenged with the HC_dose_ from the same group had virus showing that only 55.6% of the vaccinated fish were protected against post challenge virus infection using the HC_dose_ (Table 2). Put together, these data show that the LC_dose_ posed a danger of classifying less protective vaccines as highly protective against post challenge virus infection. And as such, the HC_dose_ that had ≥75% mortality (PCSP = 22.11%) in the control fish was selected for use in the next optimization steps of the cohabitation challenge model.

### Estimating the proportion of virus shedders

**Fig 2:**
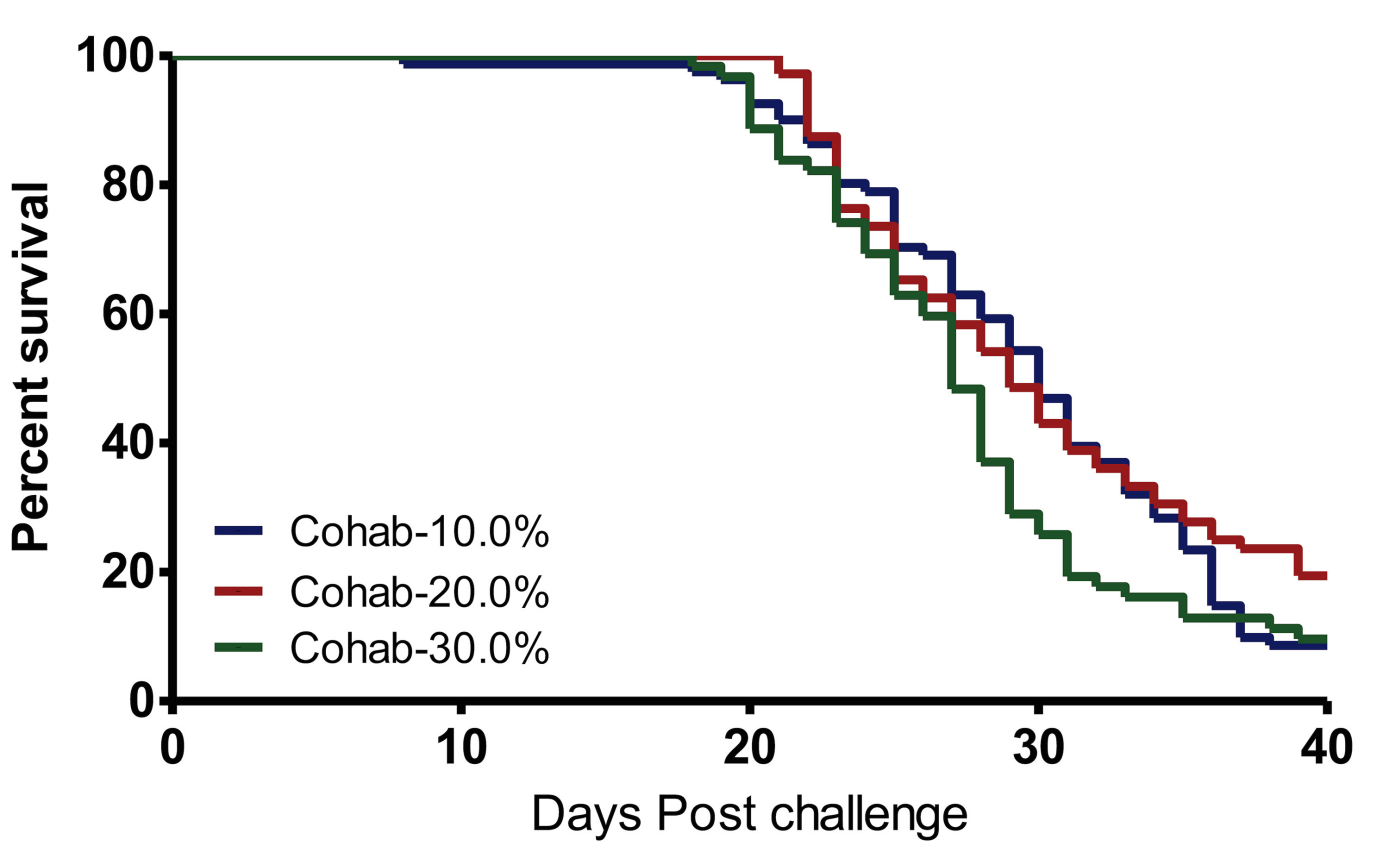
Proportion of virus shedders. KM survival analysis for cohabitees challenged with different (12.5%, 20% and 30%) proportions of virus shedders of the total number of fish per tank (*N* = 90; Study III). All virus shedders were injected with 1x10^7^ TCID_50_/fish with strain NVI015-TA. Mortality of shedders in the 10% and 30% proportion groups started on day 18 after challenge while for the 20% group day 21 after challenge was the first day of mortality. Overlaps in the KM survival curves for the cohabitees show that there was no statistical difference between the 10% and 20% (p>0.368), as well as between the 10% and 30% (p>0.1051) proportion of virus shedders of the total number of fish per tank.

Data in Fig 2 show that there was no significant difference in mortality observed in the cohabitees between the 10% and 20% (p>3.368) as well as between the 10% and 30% (p>0.1051) proportions of virus shedders used per total number of fish per tank. In addition, there was no difference on the onset of mortality observed in all three groups given that mortality in the 10% and 30% proportions of virus shedders started on day 18 post challenge while mortality in the 20% group started on day 21 post challenge (Fig 2). Overall, these data show that increasing the proportion of virus shedders to the total number of fish per tank did not influence the increase in post challenge mortality in the cohabitees and that it had no influence on the onset of mortality. All three proportions of virus shedders produced mortality >75% in the cohabitees. Consequently, a virus shedder proportion of 10% of the total number of fish per tank, which requires less fish than the 20% and 30% proportions and is in line with the 3R requirements [20], was selected for optimization of the challenge model. However, taking into account the risk of fish dying due to unforeseeable reasons such as mortality caused by handling stress when injecting the shedders with virus we increased the proportion of virus shedders from 10% to 12.5% of the total number of fish per tank for use in our challenge model.

### Susceptibility of study fish

**Fig 3:**
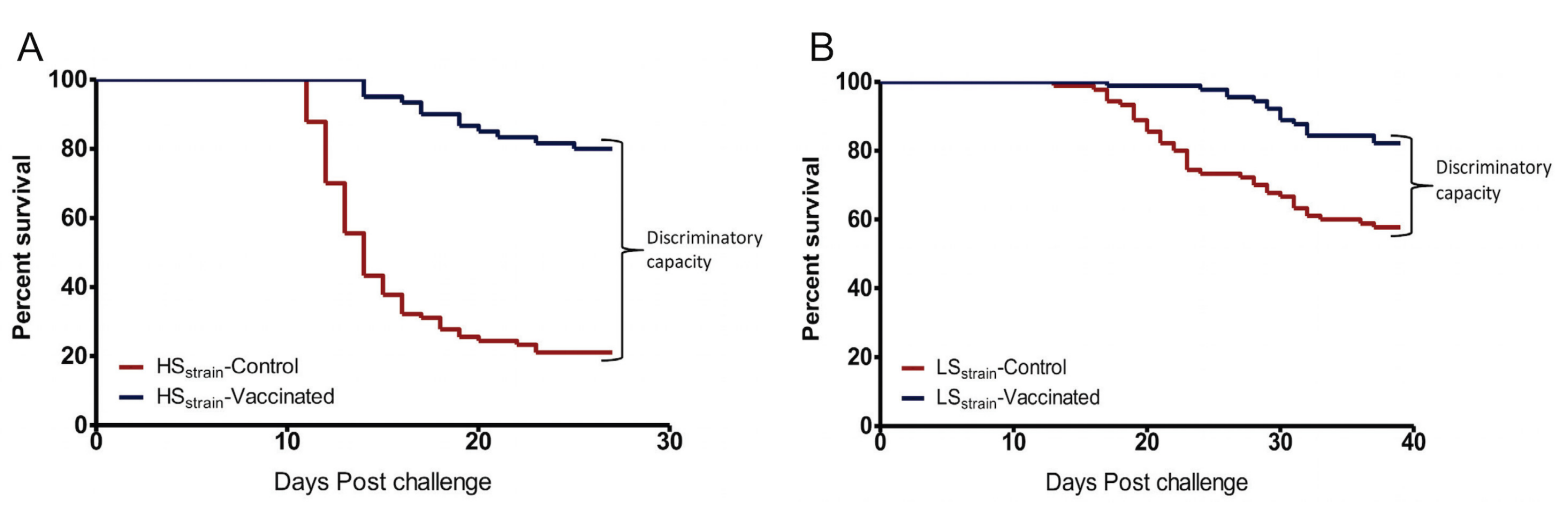
Effect of genetic susceptibility. KM survival analysis for different strains of Atlantic salmon having different susceptibility to IPNV infection, challenged with strain NVI015-TA at 1x10^7^TCID_50_/fish. (A) KM survival analysis for the highly susceptible strain (HS_strain_). (B) KM survival analysis for the less susceptible strain (LS_strain_), both including vaccinated and unvaccinated control fish. Note that the discriminatory capacity (DC) between the vaccinated and control fish for the HS_strain_ shown in Fig 3A (DC = 58.89%) was more than twice that of the LS_strain_ shown in Fig 3B (DC = 24.22%).

To evaluate the importance of using susceptible fish in optimizing the cohabitation challenge model, two susceptible strains of Atlantic salmon obtained from AquaGen (Norway), one generated from a highly susceptible strain (HS_strain_) and another from a less susceptible strain (LS_strain_), were used to select the fish strain for use in the challenge model. Although the difference in PCSP between the HS_strain_ and LS_strain_ was only 2.22% in the vaccinated fish, The PCSP for the LS_strain_ (PCSP = 57.78%, *N* = 90) was more than twofold (2.7 times) higher than PCSPs for the HS_strain_ (PCSP = 21.11%, *N* = 90) in the unvaccinated fish. Consequently, the discriminatory capacity (DC) for PCSPs between the vaccinated and control fish for the HS_strain_ (58.89%, *N* = 90) was more than twofold (2.3 times) higher than the LS_strain_ (24.22%, *N* = 90) (Table 3 and Fig 3). In addition, the risk of the HS_strain_ (HR = 5.89%, 95%CI 3.72–9.35%) dying was twofold (2.03 times) higher than the LS_strain_ (HR = 2.90%, 95%CI 1.68–4.98%). Combined, these data show that the HS_strain_ (PCSP = 21.11%) had >75% mortality in the control fish while the LS_strain_ had 42.22% (PCSP = 57.78%) mortality in the controls. Consequently, the HS_strain_ that had ≥75% mortality in the control fish and a higher DC between the vaccinated and control fish was chosen for optimization of the challenge model.

**Table 3:** Comparison of post challenge survival proportions and risk ratios of the high and low Atlantic salmon susceptible strains to NVI015-TA.

Test variables	Parameters	Less susceptible strain (LS_strain_)	Highly susceptible strain (HS_strain_)	Comparative differences between HC_dose_ and LC_dose_
Post challenge survival proportions (PCSP)	Vaccinated PCSP	82.22%(*N* = 45)	80.00%(*N* = 45)	2.22%
	Unvaccinated PCSP	57.78%(*N* = 45)	21.11%(*N* = 45)	36.67%
Discriminatory capacity (DC)	DC	24.22%	58.89%	34.67%
Hazard risk ratio	HR	2.90	5.89	2.99
	95%CI	1.68%–4.98%	3.72%–9.35%	
	P-value	P<0.000	P<0.000	

PCSP = Post challenge survival proportions; DC = discriminatory capacity calculated as DC = PCSP(vaccinated fish–unvaccinated fish); HR = Hazard ratio (risk ratio); *N* = total number of fish.

### Establishing the sampling time-points

**Fig 4:**
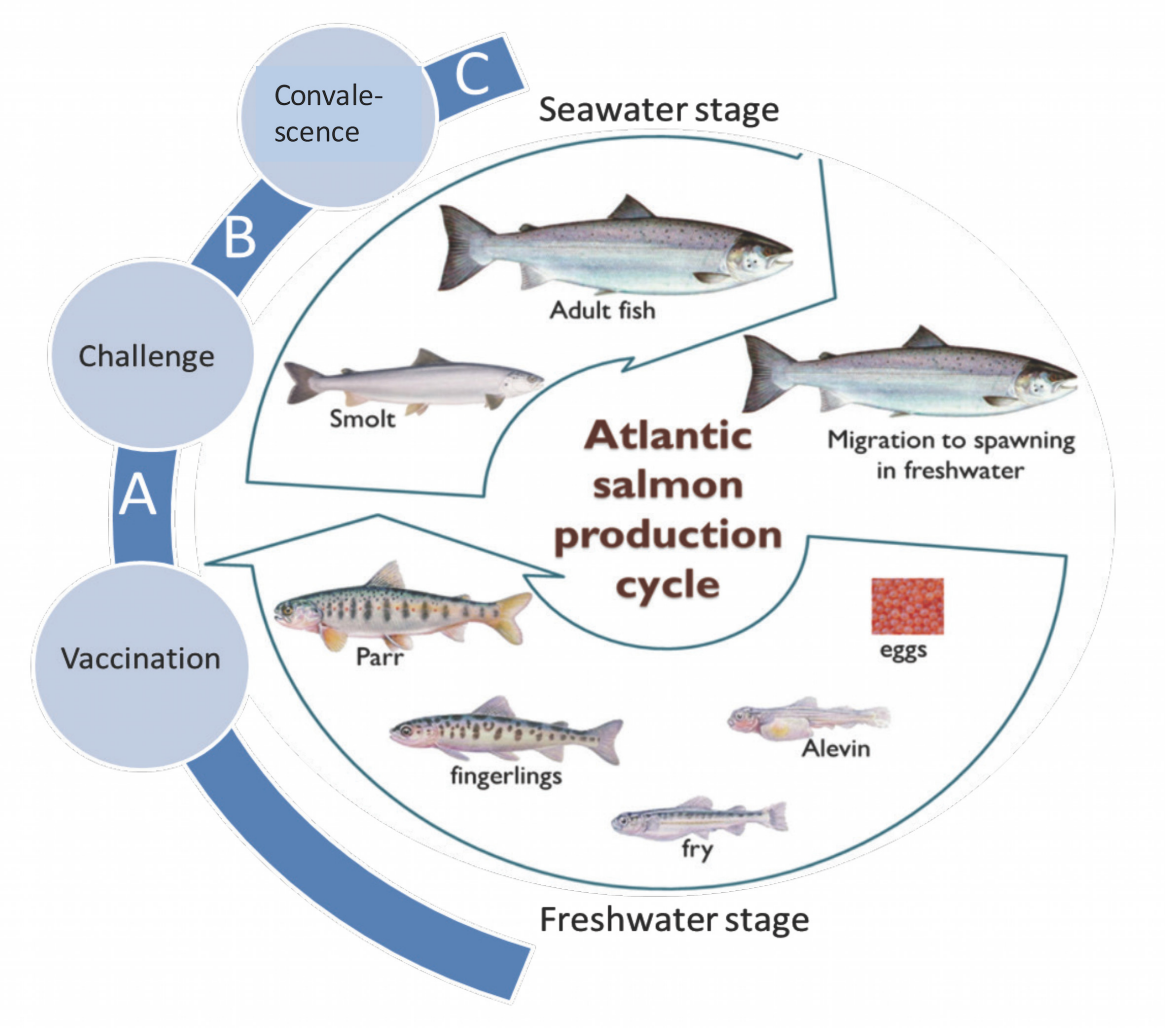
Salmon production cycle. The production cycle of Atlantic salmon depicting the timing of IPN vaccination, challenge and convalescence. **A** indicates the immune induction period being the period between vaccination and challenge. Note that vaccination is carried out at the parr stage in freshwater before smoltification. **B** indicates the time of challenge at the post-smolt stage soon after transfer to seawater, which conforms to the time when most outbreaks occur during the Atlantic salmon production cycle. **C** shows the period after lethal challenge when the survivors become carriers or persistently infected with post challenge virus during convalescence.

**Fig 5:**
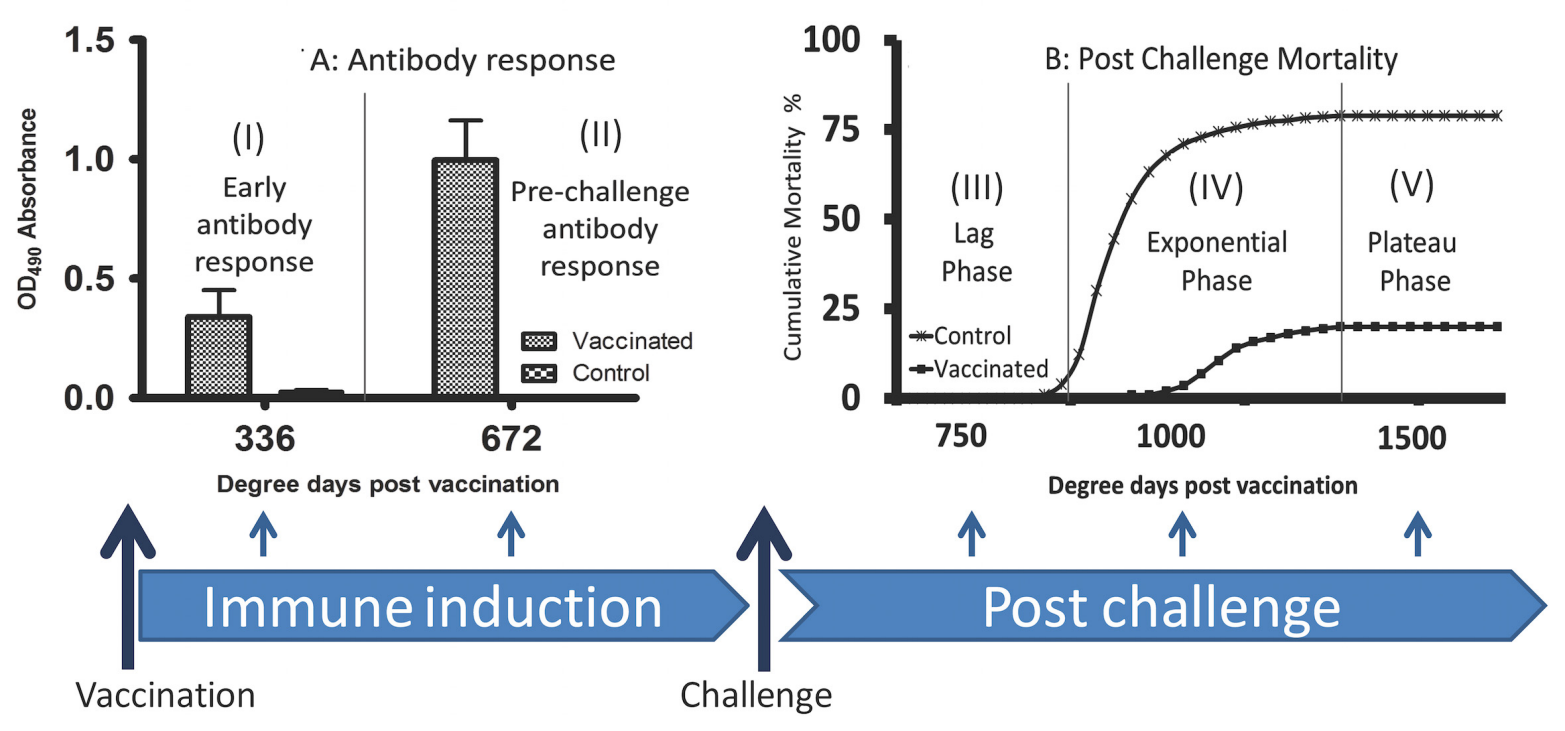
Experimental timing. Sampling time-point established for the cohabitation challenge model segmented into A and B. **(A)** shows the immune induction period (I and II), between vaccination and challenge, depicting the progression of antibody responses for fish immunized using a commercial vaccine (Pharmaq AS). Note that antibody responses increased by threefold from (I) during the early antibody responses when levels were at 0.326 OD_490_ at 330 degree days (dd) to (II) pre-challenge antibody responses when levels were at 1.221 OD_490_ at 672 dd. **(B)** shows the post challenge period depicting the progression of cumulative mortality in the vaccinated and control fish. Cumulative mortality for both the vaccinated and control fish progressed in three stages namely (III) lag-phase, (IV) exponential phase, and (V) plateau phase. Blue arrows below show the sampling time-points during immune induction and the post challenge period.

This part of the study was designed to determine the sampling time-points in conformity to the timing of vaccination and occurrence of IPN outbreaks in the production cycle of Atlantic salmon for which the challenge model was being developed. Vaccination was carried out at the parr stage before smoltification (Fig 4) while challenge was carried out at the post-smolt stage to conform with the timing when outbreaks occur at sea soon after transfer from freshwater. As shown in Fig 5, two sampling time-points were established during and at the end of the immune induction (Fig 5A) period with the first being around 336 degree days (dd) when antibody levels were at 0.340 OD_490_ (n = 12, Fig 5A, I) increasing by threefold to 0.998 OD_490_, (n = 12) at the end of the immune induction period around 670 dd post vaccination (Fig 5A, II). Antibody levels detected at the end of the immune induction period corresponded with low mortality (20%, *N* = 90) for the vaccinated fish while control fish that had no antibodies had mortality > 78.00% (*N* = 90).

To establish the sampling time-points after challenge, we followed the progression of post challenge mortality in fish challenged using the HC_dose_ defined in Study II. As shown in Fig 5B, post challenge mortality progressed in three stages with the first stage showing a lag-phase (phase III, Fig 5B) made of the incubation period when no mortality occurred, which was followed by the exponential phase (phase IV, Fig 5B) when fish started dying during acute infection. The final stage showed a plateau phase (phase V, Fig 5B) when fish stopped dying during convalescence. These three phases formed the basis for post challenge sampling time-points in which the lag-phase was used to evaluate establishment of post challenge infection during the incubation period while the exponential phase was used to evaluate the pathology.The plateau phase was used to assess the post challenge virus carrier state in the survivors during convalescence. In summary, Fig 5 shows the established sampling time points with segment A depicting the immune induction period for evaluating immune responses to vaccination while segment B the post challenge period for evaluating post challenge infection progression and mortality.

### Establishing the study design

**Fig 6:**
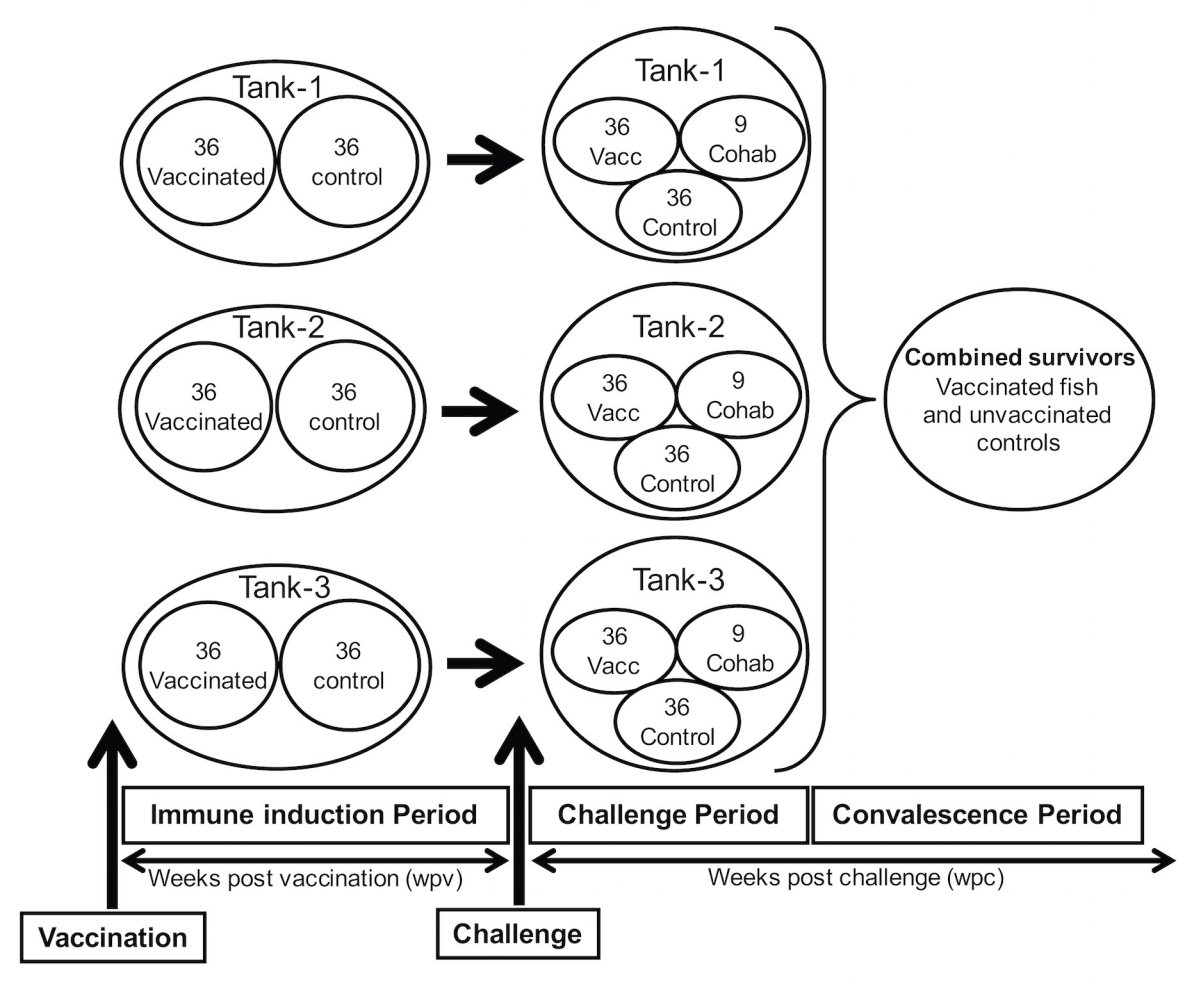
Cohabitation challenge model design. Shows a schematic design of a cohabitation challenge model based on a three parallel tank system with the number of fish estimated using the Cox PH regression model in which the statistical power function for sample size estimate was set at 80%, 95% CI and hazard risk (HR) ratio set 0.5. In each tank, 36 fish were vaccinated using a commercial vaccine (PHARMAQ AS, Oslo) and another 36 were injected with phosphate buffered saline (PBS) to serve as unvaccinated controls. After vaccination fish were kept at 12°C and left for smoltification. The study design was divided into immune induction, challenge period and convalescence stages. After smoltification, challenge was carried out by adding 9 virus shedders per tanks, which is 12.5% of the total number of fish per tank, and the virus shedders are injected with 1 x 10^7^ TCID_50_/fish of strain NVI015-TA. The immune induction period was for evaluating immune responses prior to challenge while the challenge period enables evaluation of PCSP/RPS. The convalescence period covers the period after acute infection when fish stopped dying and enabled evaluation of post challenge recovery as well as assessing the number of virus carriers linked to persistent infections.

**Fig 7:**
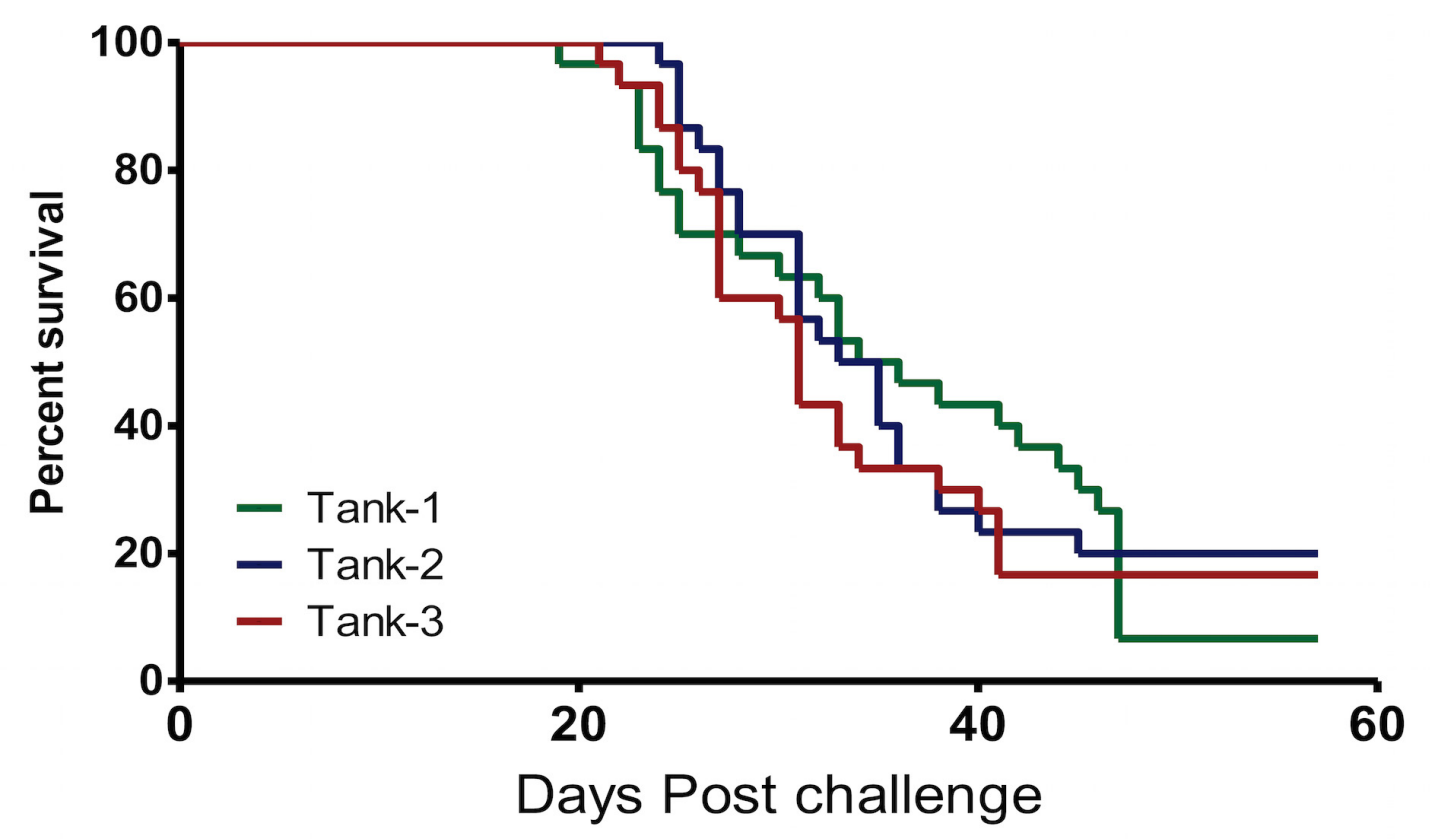
Mortality in non-vaccinated controls. KM survival analysis for comparison of PCSP induced by strain NVI015-TA in the control fish challenged using a three parallel tank system described in Fig 6. Mortality in Tanks 1, 2 and 3 started at 19, 24, and 21 days post challenge, respectively. Overlaps in the KM survival curves for control fish from Tanks 1, 2 and 3 show that there was no statistical difference (p = 0.8223) in PCSPs for the three tanks.

**Fig 8:**
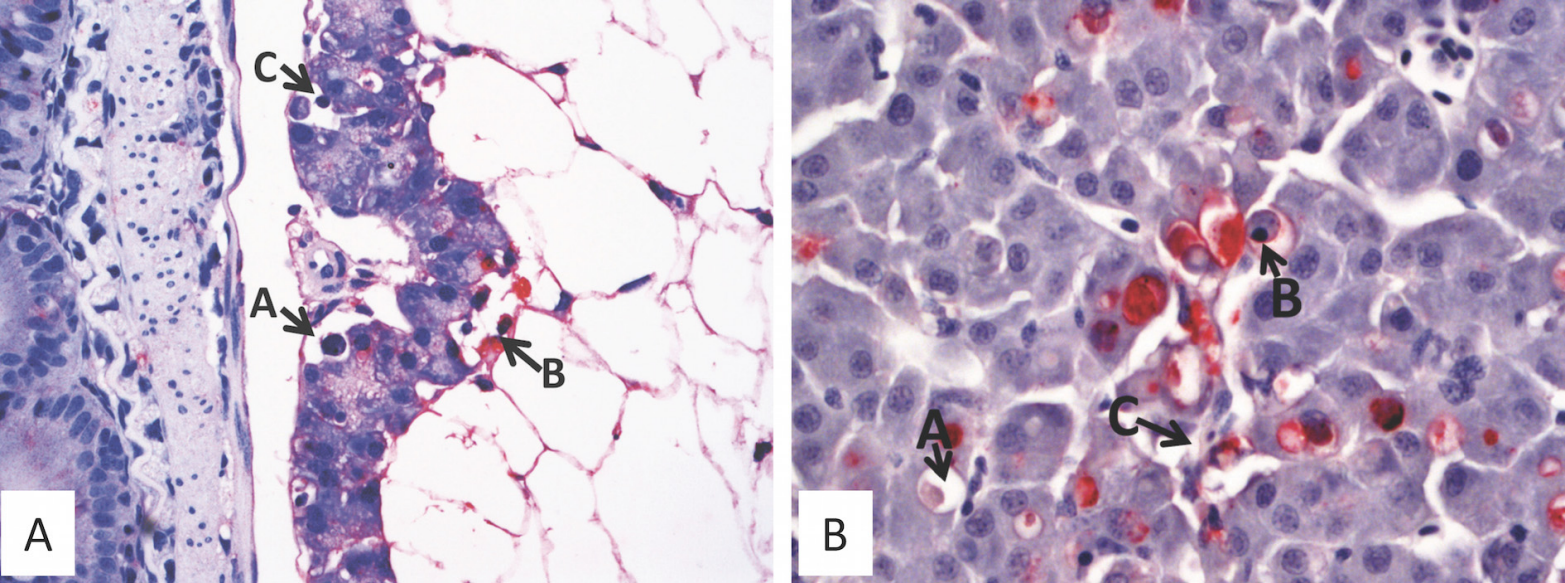
Immunohistochemistry of target organs. Immunohistochemistry (IHC) of fish infected with strain NVI015-TA. Red stain shows presence of IPNV in the infected tissues. (A) Exocrine pancreas with condensed and pyknotic nuclei (A,B,C) are indicative of cellular degeneration and necrosis. (B) Liver with vacuolation (A) and cells with apoptotic nuclei (B) and necrotic liver cells (C).

Sample size estimates using the Cox HP regression model showed that a total of 72 fish subdivided into 36 vaccinated and 36 unvaccinated control fish were required per tank. As a result, each of the three parallel tanks was allocated 72 fish as shown in Fig 6. After challenge, individual PCSP for the three tanks were Tank-1 = 93.33%, Tank-2 = 80.33% and Tank-3 = 80.00%. The mean PCSP for the three tanks was estimated at 84.57% (n = 3; Fig 7). After challenge, the mean PCSP for the three tanks was estimated at 82.5% (n = 3, SD = 7.60%). The risk of control fish dying in the three tanks showed that there was no significant difference (p = 0.8223) among the three tanks indicating that fish challenged using strain NVI015-TA had the same risk of dying in all the tanks. During acute infection and mortality pathology was seen in exocrine pancreas (Fig 8A) and in liver parenchyma (Fig 8B). Put together these data show that the use of a three parallel tank system can be used to check the consistency of the model during lethal challenge by producing similar results from three independent tanks subjected to same input variables. As shown in Fig 6, survivors of lethal challenge at the end of the post challenge period were pooled into one tank because data obtained from the convalescent stage was not used to evaluate vaccine efficacy based on lethal challenge, but were used to evaluating the post challenge virus infection rate as shown in section 3.2 above.

### Testing the reproducibility of the model

Reproducibility was defined as the ability of the challenge model to generate similar results when tested at least three times using similar optimized input variables enlisted below.

1. *Challenge virus*; strain NVI015-TA (Section 3.1 above, Table 1)
2. *Challenge dose*; 1 x 10^7^ TCID_50_/fish (Section 3.2, Table 2)
3. *Virus shedders proportion*; 12.5% of total number of fish per tank (Section 3.3, Fig 3)
4. *Study fish*; Standard bred Atlantic salmon susceptible to IPN (Section 3.4, Table 3)
5. *Vaccination stage*; Parr (freshwater stage) (Section 3.5, Fig 4)
6. *Challenge stage*; Post-smolts (seawater stage) (Section 3.5, Fig 4)
7. *Sampling time-points*; Immune induction and post challenge (Section 3.5, Fig 4)
8. *Study design*; Three parallel tanks system (Section 3.6, Fig 5)

Data in Table 4 shows that the model was reproducible by generating similar results for four cohabitation challenge trials carried out using similar input variables in a two and three parallel tank system. It is interesting to note that cumulative mortality in the unvaccinated control fish for the three independent vaccine efficacy trials shown in Table 4 was maintained between 84.6% and 86.3%, which was above the cutoff limit ≥75% set for optimization of the challenge model. Although the vaccines batches used in the study were different, the DC between the vaccinated and control fish was maintained at ≥60% for all three independent vaccine efficacy trials. In terms of inter-tank variation, it is interesting to note that both the two and three parallel tank systems had no significance difference in the mean PCSPs for the control fish in each trial indicating that fish in all tanks had the same risk of dying. This was supported by the low inter-tank standard deviation (STDEV) (<8.0%) of the mean PCSP for control fish in each trial. In summary, data in Table 4 shows that the model is reproducible by consistently producing similar results from three independent vaccine efficacy trials with PCSP, STDEV and DC for the unvaccinated control fish maintained at ≤12%, <8.0% and >65%, respectively.

**Table 4:** Comparison of three independent vaccine efficacy trials using the cohabitation challenge model in Atlantic salmon parr.

Parameters	Project Code		
	H1462	H1304	H3008
Total number of fish	202	222	228
Number of parallel tanks	2	2	3
Total vaccinated fish*	98	110	114
Total unvaccinated control fish	104	112	114
Average number of fish allocated to each group per tank	49–52	55–56	38
Mean PCSP vaccinated fish	74.4%(*N* = 98)	80.9% (*N* = 110)	89.5% (*N* = 114)
Mean PCSP unvaccinated control	14.4%(*N* = 104)	13.7%(*N* = 112)	15.4% (*N* = 114)
Mean cumulative mortality in unvaccinated control group	85.6%(*N* = 104)	86.3% (*N* = 112)	84.6% (*N* = 114)
Discriminatory capacity (DC)	60.02%	67.2%	74.1%
Significance of inter-tank PCSP variation in control tanks	P = 0.6072	P = 0.9953	P = 0.8223
Standard deviation of the mean PSCP for the control tanks	1.36%	5.0%	7.6%

PCSP = Post challenge survival proportions; *N* = number of fish.

*Note that different vaccine batches were used.

## Discussion

In vaccine efficacy, the reliability of a challenge model can be defined as the ability of the model to effectively discriminate vaccine-protected fish from the unvaccinated control fish. Hence, the wider the DC between the vaccinated fish and control fish the higher the reliability of the model. To attain this, Amend [21] recommended 60% post challenge mortality in control fish to allow for adequate discrimination between the vaccinated and control fish. Although we are in agreement with Amend’s threshold limit of 60% cumulative mortality in the unvaccinated controls [21], to further increase the DC between the vaccinated and control fish of our model we raised the cutoff limit of post challenge mortality in the control fish to ≥75%. And as such, we used a targeted approach to individually optimize all input variables to attain the established benchmark of ≥75% mortality in the control fish. Consequently, the overall output when summed up resulted in a cohabitation challenge model that attained ≥75% mortality in the unvaccinated control fish, which increased the DC of our model to effectively differentiate vaccine protected fish from control fish as a measure of vaccine efficacy. Put together, data presented here shows that our model is reliable and that it is reproducible by generating similar results in four challenge trials using the same input variables.

The first and foremost input variable identified in the present study that determines the outcome of the *in vivo* challenge was the challenge virus strain. The virulent strain has the capacity to produce high mortality and results in development of pathognomonic features of IPN in susceptible fish. As pointed out in our previous studies [4], this input variable is a prerequisite for efficacy evaluation of IPN vaccines in terms of protection against mortality, its ability to prevent post challenge infection and pathology in vaccinated fish. We have shown the importance of the T_217_A_221_ motif as a marker virus virulence for IPNV in Atlantic salmon, corroborating previous studies (Santi et al. 2004, Song et al. 2005). We have shown that viruses encoding this motif have a wide DC between the vaccinated and control fish by yielding <15% PCSP in control fish. Conversely, fish infected with strains encoding the P_217_T_221_ motif had low mortality and had an insignificantly low risk of dying which is in line with our previous findings showing that the P_217_T_221_ motif was linked to subclinical infections that do not cause mortality in infected fish while the T_217_A_221_ motif was linked to clinical disease and high mortality in Atlantic salmon [22].

The second important input variable was the challenge dose. A low challenge dose poses the danger of classifying less protective vaccines (sub-potent) as highly protective, which could compromise the reliability of the challenge model. Vaccinated fish were shown to be 100% protected against post challenge infection when challenged using the LC_dose_ and yet fish from the same group had an infection rate of 44.4% when challenged using the HC_dose_ showing that the LC_dose_ posed a danger of classifying a less protective vaccine batches as protective. We have also shown that the LC_dose_ caused low mortality both in the vaccinated and control fish. And as such, the HC_dose_ had a twofold higher DC for PCSPs than the LC_dose_ between the vaccinated and control fish. Given that the reliability of a challenge model is determined by its degree to effectively differentiate the level of protection between batches of different potency, and particularly identifying sub-potent batches, it follows that a challenge dose that produces a wide DC is more reliable in differentiating between potent and sub-potent vaccine batches than a challenge dose that produces a low DC. Therefore, it can be concluded from our findings that a challenge dose of 1 x 10^7^ TCID_50_/fish for strain NVI015-TA gives a better DC than the 1 x 10^5^ TCID_50_/fish challenge dose. Overall, these findings show that the ability of a challenge model is not only dependent on selecting a highly virulent challenge virus strain, but is also dependent on the challenge dose.

Another important factor identified in the optimization of the cohabitation challenge model for IPNV in Atlantic salmon was determination of the proportion of virus shedders to serve as a source of infection to the cohabitees. Various proportions of virus shedders have been used in cohabitation models for IPNV. For example, Bowden et al [23] used an equal proportion of shedders to the total number of fish per tank injected with 1 x 10^7^ TCID_50_/fish of challenge virus in which they obtained 60% mortality in the controls being lower than the ≥75% mortality obtained in the current study despite using a higher proportion of shedders than what was used in our study. In another study, Ramstad et al [24] used a shedder proportion of 50% (*N* = 238) and obtained 74% mortality in controls (cohabitees). Shedders were injected with 1 x10^7^ TCID_50_/mL of challenge virus encoding the T_217_A_221_ motif [25]. In the present study there was no significant difference in PCSP for virus-shedder proportions of 10%, 20% and 30% of the total number of fish per tank suggesting that increasing the proportion of virus shedders did not result in increased post challenge mortality in the cohabitees. And as such, a 12.5% virus shedder proportion that requires fewer fish than the 20% and 30% proportions was considered sufficient for fish challenged using strain NVI015-TA in a cohabitation challenge model. Overall, these findings suggest that IPNV is highly infectious and that few fish are required for transmission between virus shedders and susceptible fish. However, there is need for detailed transmission studies to consolidate these observations in Atlantic salmon.

The susceptibility of fish used for developing a challenge model has a significant influence on the outcome of the model. Ramstad and Midtlyng [26] showed that post-smolts derived from parent stocks that were highly susceptible to IPNV produced high mortality >75% while post-smolts from less susceptible parents had low mortality varying between 26% and 35%. In their findings they noted that control fish derived from IPNV susceptible parents had a better capacity to show significant differences in levels of protection between vaccinated and control fish while less susceptible fish could not produce sufficient differences in protection levels between vaccinated and control. Based on these observations, Ramstad and Midtlyng [26] concluded that mortality in control fish should exceed 50% to reliably show the level of protection in fish vaccinated against IPNV. In line with their observations, our findings show that less susceptible fish having a mortality of 42% in the controls had a low DC between the vaccinated and control fish compared to fish that had ≥75% mortality in the controls that had a high DC indicating that the susceptibility of fish used to establish a challenge model has a significant influence on the outcome of the model.

It is important that the design of the challenge model conforms to standard procedures used for the evaluation of vaccine efficacy for finfish [12]. As pointed out by the European Medicine Agency (EMA) [12], the sample size of fish allocated for vaccine efficacy trials should be sufficient to allow the results to be statistically significant and clinically reliable [27–29]. Hence, in the present study, we used the Cox HP regression model to determine the sample size that would yield significant differences between the vaccinated and control fish as well as to determine the relative risk of fish dying after exposure to strain NVI015-TA in a cohabitation challenge model. To our knowledge, this is the first study design that uses a statistical approach to estimate the sample size of fish required to showing statistical differences in levels of protection between the vaccinated and control fish in a cohabitation challenge model for IPNV. Another important factor pointed out by EMA [12] is that a minimum of two parallel tanks should be used for each vaccine group to allow for statistical evaluation of inter-tank variations as a measure of reproducibility of the model. In compliance with this recommendation, we used a three parallel tank system in the design of our challenge model although data presented here shows that the model is also reproducible with a two parallel tank system so long all optimized input variables are kept constant. Overall, our findings show that our model is reliable and reproducible by maintaining inter-tank variations <8.0% and mortality ≥75%, which is in line with, if not better than observations made by Amend [30] who recommended an inter-variation of <20% and mortality ≥ 60% in control fish.

In summary, this study shows that a reliable and reproducible cohabitation challenge model can be developed for evaluating the efficacy of IPNV vaccines in Atlantic salmon. We have shown that developing an effective cohabitation challenge model is a stepwise process that demands for identifying and optimizing different input variables required to build the model. By keeping the input variables constant, we have shown that an optimized cohabitation challenge model for IPN has the capacity to effectively differentiate vaccine-protected fish from control fish as a measure of efficacy and that it can be used to compare the efficacy of different formulations of vaccines produced using different antigen delivery systems as shown in our previous studies [6]. Overall, we envisage that the approach used in this study shall open new avenues for developing optimal challenge models for use in evaluating the efficacy of different fish vaccines used in aquaculture.

## References

[1] Dobos P (1976) Size and Structure of Genome of Infectious Pancreatic Necrosis Virus. Nucleic Acids Research 3: 1903–1924. 987579 10.1093/nar/3.8.1903 PMC343048

[2] Jarp J, Gjevre AG, Olsen AB, Bruheim T (1995) Risk-Factors for Furunculosis, Infectious Pancreatic Necrosis and Mortality in Post-Smolt of Atlantic Salmon, Salmo-Salar l. Journal of Fish Diseases 18: 67–78.

[3] Gomez-Casado E, Estepa A, Coll JM (2011) A comparative review on European-farmed finfish RNA viruses and their vaccines. Vaccine 29: 2657–2671. 10.1016/j.vaccine.2011.01.097 21320546

[4] Munang'andu HM, Mutoloki S, Evensen O (2014) Acquired immunity and vaccination against infectious pancreatic necrosis virus of salmon. Developmental and Comparative Immunology 43: 184–196. 10.1016/j.dci.2013.08.008 23962742

[5] Munang'andu HM (2013) Vaccinology of infectious pancreatic necrosis virus: Immunogenicity, signatures of infection and correlates of protective immunity. PhD Thesis, Norwegian School of Veterinary Science, Oslo, Norway.

[6] Munang'andu HM, Fredriksen BN, Mutoloki S, Brudeseth B, Kuo TY, Marjara IS, Dalmo RA, Evensen O (2012) Comparison of vaccine efficacy for different antigen delivery systems for infectious pancreatic necrosis virus vaccines in Atlantic salmon (Salmo salar L.) in a cohabitation challenge model. Vaccine 30: 4007–4016. 10.1016/j.vaccine.2012.04.039 22537985

[7] Munang'andu HM, Fredriksen BN, Mutoloki S, Dalmo RA, Evensen O (2013) The kinetics of CD4+and CD8+T-cell gene expression correlate with protection in Atlantic salmon (Salmo salar L) vaccinated against infectious pancreatic necrosis. Vaccine 31: 1956–1963. 10.1016/j.vaccine.2013.02.008 23422142

[8] Munang'andu HM, Fredriksen BN, Mutoloki S, Dalmo RA, Evensen O (2013) Antigen dose and humoral immune response correspond with protection for inactivated infectious pancreatic necrosis virus vaccines in Atlantic salmon (Salmo salar L). Veterinary Research 44. 10.1186/1297-9716-44-7 PMC3668999 23398909

[9] Munang'andu HM, Sandtro A, Mutoloki S, Brudeseth BE, Santi N, Evensen O (2013) Immunogenicity and Cross Protective Ability of the Central VP2 Amino Acids of Infectious Pancreatic Necrosis Virus in Atlantic Salmon (Salmo salar L.). Plos One 8. 10.1371/journal.pone.0054263 PMC3549989 23349841

[10] Santi N, Vakharia VN, Evensen O (2004) Identification of putative motifs involved in the virulence of infectious pancreatic necrosis virus. Virology 322: 31–40. 15063114 10.1016/j.virol.2003.12.016

[11] Bowden TJ, Smail DA, Ellis AE (2002) Development of a reproducible infectious pancreatic necrosis virus challenge model for Atlantic salmon, Salmo salar L. Journal of Fish Diseases 25: 555–563.

[12] EMEA (1998) European Agency for the Evaluation for Medicinal Products CVMP/VICH/590/98-Final: Guideline on validation of analytical procedures: definition and terminology. London, UK.

[13] Blackwelder WC (1993) Sample-Size and Power for Prospective Analysis of Relative Risk. Statistics in Medicine 12: 691–698. 8511445 10.1002/sim.4780120708

[14] Carpenter TE (2001) Use of sample size for estimating efficacy of a vaccine against an infectious disease. American Journal of Veterinary Research 62: 1582–1584. 11592323 10.2460/ajvr.2001.62.1582

[15] Rao MR, Blackwelder WC, Troendle JF, Naficy AB, Clemens JD (2002) Sample size determination for phase II studies of new vaccines. Vaccine 20: 3364–3369. 12213406 10.1016/s0264-410x(02)00317-1

[16] Evensen O, Olesen NJ (1997) Immunohistochemical detection of VHS virus in paraffin-embedded specimens of rainbow trout (Oncorhynchus mykiss); The influence of primary antibody, fixative, and antigen unmasking on method sensitivity. Veterinary Pathology 34: 253–261. 9163887 10.1177/030098589703400316

[17] Evensen O, Lorenzen E (1997) Simultaneous demonstration of infectious pancreatic necrosis virus (IPNV) and Flavobacterium psychrophilum in paraffin-embedded specimens of rainbow trout Oncorhynchus mykiss fry by use of paired immunohistochemistry. Diseases of Aquatic Organisms 29: 227–232.

[18] Kaplan EL, Meier P (1958) Nonparametric-Estimation from Incomplete Observations. Journal of the American Statistical Association 53: 457–481.

[19] Cox DR (1972) Regression Models and Life-Tables. Journal of the Royal Statistical Society Series B-Statistical Methodology 34: 187–+.

[20] Rusche B (2003) The 3Rs and animal welfare—conflict or the way forward? ALTEX 20: 63–76. 14671703

[21] Amend A (1981) Potency testing of fish vaccines. Dev Biol Stand 49: 447–450.

[22] Mutoloki S, Munang'andu HM, Evensen O (2013) Clinical and subclinical forms of infectious pancreatic necrosis virus infections show specific viral genetic fingerprints that link differences in virulence to immunogenicity. Fish & Shellfish Immunology 34: 1667.

[23] Bowden TJ, Lockhart K, Smail DA, Ellis AE (2003) Experimental challenge of post-smolts with IPNV: mortalities do not depend on population density. J Fish Dis 26: 309–312. 12962240 10.1046/j.1365-2761.2003.00456.x

[24] Ramstad A, Romstad AB, Knappskog DH, Midtlyng PJ (2007) Field validation of experimental challenge models for IPN vaccines. Journal of Fish Diseases 30: 723–731. 18034679 10.1111/j.1365-2761.2007.00858.x

[25] Santi N, Vakharia VN, Evensen O (2004) Identification of putative motifs involved in the virulence of infectious pancreatic necrosis virus. Virology 322: 31–40. 10.1016/j.virol.2003.12.016 S0042682204000121 [pii]. 15063114

[26] Ramstad A, Midtlyng PJ (2008) Strong genetic influence on IPN vaccination-and-challenge trials in Atlantic salmon, Salmo salar L. Journal of Fish Diseases 31: 567–578. 10.1111/j.1365-2761.2008.00929.x 18482384

[27] Blackwelder WC (1993) Sample-Size and Power for Prospective Analysis of Relative Risk. Statistics in Medicine 12: 691–698. 8511445 10.1002/sim.4780120708

[28] Carpenter TE (2001) Use of sample size for estimating efficacy of a vaccine against an infectious disease. American Journal of Veterinary Research 62: 1582–1584. 11592323 10.2460/ajvr.2001.62.1582

[29] Rao MR, Blackwelder WC, Troendle JF, Naficy AB, Clemens JD (2002) Sample size determination for phase II studies of new vaccines. Vaccine 20: 3364–3369. 12213406 10.1016/s0264-410x(02)00317-1

[30] Amend A (1981) Potency testing of fish vaccines. Dev Biol Stand 49: 447–450.

